# Trends of Blood Lead Levels in US Pregnant Women: The National Health and Nutrition Examination Survey (2001–2018)

**DOI:** 10.3389/fpubh.2022.922563

**Published:** 2022-07-01

**Authors:** Jing Wang, Yujie Yang, Juan Zhang, Na Liu, Huifang Xi, Hong Liang

**Affiliations:** ^1^Jinan Maternity and Child Care Hospital Affiliated to Shandong First Medical University, Jinan, China; ^2^The First Affiliated Hospital of Shandong First Medical University, Jinan, China

**Keywords:** blood lead levels, lead exposure, pregnancy, prevalence, trend

## Abstract

**Objectives:**

This study investigates the trends of blood lead levels in US pregnant women based on the National Health and Nutrition Examination Survey from 2001 to 2018.

**Methods:**

A total of 1,230 pregnant women were included in this study. The weighted logistic regression was applied to analyze the association between sociodemographic characteristics with high blood levels. We computed the blood lead levels for each survey period from 2001–2002 to 2017–2018. Moreover, we used the adjusted linear regression model to investigate the time-related change in blood lead level. The odds ratio (OR) with a 95% confidence interval (CI) was calculated accordingly.

**Results:**

The mean blood lead was 0.73 ± 0.03 ug/dL, and high blood lead was observed in 2.53% of individuals. The Mexican Americans were more associated with high blood lead than the non-Hispanic white (OR, 1.072; 95% CI, 1.032-1.112). The mean blood lead level has decreased from 0.97 ug/dL in 2001–2002 to 0.46 ug/dL in 2013–2014. Afterward, a slight increase was observed with the mean blood lead of 0.55 ug/dL in 2015–2016 and 0.53 ug/dL in 2017-2018. In the adjusted linear regression model, each year's increase would lead to a 0.029 ug/dL decrease in blood lead (*P* < 0.001). However, no significant change was observed in the 2017–2018 cycle compared with 2009–2010 (*P* = 0.218).

**Conclusion:**

This study summarized the trend of blood lead levels in US pregnant women over 2001–2018. Continued effort is still required to control lead sources better and protect this population from lead exposure.

## Introduction

As a widespread and persistent neurotoxin, lead exposure remains a public health concern despite the decline in environmental sources. Previous studies have revealed the adverse impact of prenatal lead exposure on maternal health, birth outcomes, and neurodevelopment (1, 2). Owning to the policies against environmental lead sources, the lead exposure of the general US population has been substantially declining over time (3, 4). However, there are no safe levels of lead exposure in pregnant women since the toxic effects were observed even in lower levels than previously identified harmful (5).

Lead can readily cross through the placenta by passive diffusion, which makes the lead concentrations in fatal highly associated with the maternal blood lead levels (6). During pregnancy, the increased bone turnover would mobilize bone lead stores and release lead into maternal blood (7, 8). Considering the decreased environmental sources, the cumulative lead in pregnant women would become an endogenous lead source making infants at a higher risk of lead exposure (9). Despite the adverse impact, blood lead levels were not routinely tested in pregnant women. It remains unclear about the trends of lead exposure in US pregnant women.

This study aims to investigate the trends of blood lead levels in US pregnant women based on the National Health and Nutrition Examination Survey (NHANES) from 2001 to 2018. The association between sociodemographic characteristics and blood lead level was also analyzed.

## Methods

### Data Source

NHANES is a cross-sectional survey conducted by the National Center for Health Statistics, which provides population-based surveillance on multiple environmental contamination exposure. NHANES has become the cornerstone of managing contamination prevention guidelines, standards, and abatement strategies. The continuous NHANES was conducted every 2 years to assess the health status of a representative sample of the non-institutionalized US population. This study collected data from 9 consecutive NHANES cycles, including 2001–2002, 2003–2004, 2005–2006, 2007–2008, 2009–2010, 2011–2012, 2013–2014, 2015–2016, 2017–2018. We included pregnant women aged 18 and above, and those without blood lead measurements were excluded. Finally, a total of 1,230 individuals were analyzed in this study.

### Patient and Public Involvement

NHANES is a cross-sectional survey conducted by the National Center for Health Statistics, which provides population-based surveillance on multiple environmental contamination exposure. NHANES was approved by the National Center for Health Statistics Research Ethics Review Board (NCHS Research Ethics Review Board Approval Protocol #98-12; Protocol #2005-06; Protocol #2011–17). Written informed consent was acquired from all individuals.

### Blood Lead Measurement

National Center for Health Statistics provides detailed instructions on blood specimen collection and processing in the NHANES Laboratory Procedures Manual (https://wwwn.cdc.gov/nchs/data/nhanes/2013-2014/manuals/2013_mec_laboratory_procedures_manual.pdf). The analysis on whole blood specimens was performed in the Division of Laboratory Sciences, National Center for Environmental Health, and Centers for Disease Control and Prevention. Samples were appropriately stored under frozen conditions (−20°C) until testing. Blood lead was measured in the 2001–2002 cycle using a PerkinElmer Model SIMAA 6000 simultaneous multielement atomic absorption spectrometer (https://wwwn.cdc.gov/Nchs/Nhanes/2001-2002/L06_B.htm). Since the 2003–2004 cycle, the lead level of whole blood has been determined using inductively coupled plasma mass spectrometry-based on quadrupole ICP-MS technology. Iridium (192.963) was set as the internal standard for lead (207.977) with a dwell time of 100 ms and RPq value of 0.25. The dilution of blood specimens is a simple dilution of 1 part sample + 1 part water + 48 parts diluent prior to analysis. The diluent is an aqueous solution of 5 μg/L internal standard mixture, in 0.4% v/v tetramethyl ammonia hydroxide, 1% ethyl alcohol, 0.01% APDC, and 0.05% v/v Triton X-100. The internal standard intermediate mixture was composed of 50 mL of 20 mg/L Rh, Ir, and Te in 1% v/v HNO_3_. Detailed methods for blood lead analysis from 2003-2004 to 2017-2018 cycle were provided on the NHANES website (https://wwwn.cdc.gov/nchs/data/nhanes/2013-2014/labmethods/PbCd_H_MET.pdf). The quality assurance and quality control protocols of NHANES followed the 1988 Clinical Laboratory Improvement Act mandates. This study defined a blood lead of >2 ug/dL as high blood lead (10, 11).

### Sociodemographic Characteristics

This study collected sociodemographic characteristics, including age, race/ethnicity, education levels, and poverty-to-income ratio (PIR). Race/ethnicity was categorized into non-Hispanic white, non-Hispanic black, Mexican American, other Hispanic, and other races. PIR was calculated by the following equation: $\frac{total\;family\;income}{federal\;poverty\;threshold\;}$, and PIR levels were stratified as <1.33, 1.33–3.5, and ≥3.5.

### Statistical Analysis

All statistical analysis was performed using R software (version 4.1.1). NHANES applied a complex multistage probability sampling design to acquire nationally representative samples. Following the recommendation of NHANES, we applied the 18-year NHANES weights (calculated by $\frac{1}{9}\times WTMEC2YR$) to adjust for the bias caused by survey non-response, non-coverage, and unequal selection probabilities. We performed all statistics considering the multistage sampling design (12, 13).

Variables were represented as weighted mean ± standard error (continuous) or proportions (categorical). Comparisons between groups (age below median and age equal or above median) applied the weighted *t*-test for continuous variables and the chi-square test for categorical variables. The weighted univariate logistic regression was used to analyze the association of age, race/ethnicity, education, PIR levels, and year cycle. The odds ratio (OR) with a 95% confidence interval (CI) was calculated.

We computed and illustrated the weighted blood lead levels and the prevalence of high blood lead for each NHANES survey period from 2001–2002 to 2017–2018. Moreover, we used the weighted linear regression model to investigate the time-related change in blood lead level. The adjusted model considered age, race/ethnicity, education, and PIR levels. *P*-value <0.05 was considered statistically significant. Additionally, we analyzed the 2-year cycles as a continuous variable to investigate the time-related change in blood lead. The smoothed curve on the association between year cycle and blood lead levels was illustrated by the svysmooth function by the “survey” package.

## Results

### Patient Characteristics

Patient characteristics are represented in Table 1. The mean age was 28.17 ± 0.29 years, and the most race/ethnicity was non-Hispanic white (52.74%). The mean blood lead was 0.73 ± 0.03 ug/dL, and high blood lead was observed in 2.53% of individuals. The elder group showed significantly higher education (*P* < 0.001) and PIR levels (*P* < 0.001). There was no significant difference in race/ethnicity, blood lead, and high blood lead between the young (age ≤ 27) and the elder (age >27) group.

**Table 1:** The characteristics of participants.

	**All**	**Age ≤27**	**Age >27**	***P***
***N***	**1,121**	**616**	**505**	
Age (years)	28.17 ± 0.29	23.32 ± 0.13	33.05 ± 0.30	<0.001
**Race/ethnicity (** ***n*** **, %)**				**0.14**
Non-Hispanic white	52.74	49.46	55.73	
Non-Hispanic black	15.49	18.76	12.87	
Mexican American	16.20	18.10	14.28	
Other Hispanic	5.55	5.93	5.30	
Other races	10.03	7.75	11.82	
**Education (** ***n*** **, %)**				<0.001
Below high school	19.45	28.64	10.80	
High school	18.89	26.08	12.13	
Above high school	61.66	45.28	77.06	
**PIR levels (%)**				<0.001
<1.33	26.36	35.82	19.15	
1.33–3.5	31.56	37.10	25.76	
≥3.5	42.07	27.08	55.09	
Blood lead (ug/dL)	0.73 ± 0.03	0.73 ± 0.04	0.73 ± 0.03	0.94
High blood lead (%)	2.53	2.72	2.39	0.78

*Weighted analysis was performed. High blood lead is defined as blood lead >2 ug/dL. PIR, poverty-to-income ratio*.

### The Association Between Sociodemographic Characteristics and High Blood Lead

The weighted univariate logistic regression showed that the year cycle was negatively correlated with the prevalence of high blood lead (OR, 0.996; 95% CI, 0.993-0.999; *P* = 0.038). Moreover, compared with the non-Hispanic white, the Mexican Americans were more likely to develop high blood lead (OR, 1.072; 95% CI, 1.032–1.112; *P* < 0.001). However, there was a significant association of age, education, and PIR levels with high blood lead prevalence. The association between sociodemographic characteristics and high blood lead is summarized in Table 2.

**Table 2:** The weighted logistic regression on the prevalence of high blood lead in pregnant females.

	**OR**	**95% CI**	***P***
Age	0.999	(0.998–1.000)	0.064
**Race/ethnicity**			
Non-Hispanic white	*Reference*		
Non-Hispanic black	1.012	(0.992–1.031)	0.24
Mexican American	1.072	(1.032–1.112)	<0.001
Other Hispanic	1.007	(0.994–1.02)	0.323
Other races	1.107	(0.992–1.235)	0.07
**Education**
Below high school	*Reference*		
High School	0.987	(0.956–1.019)	0.408
Above high school	0.988	(0.958–1.019)	0.448
**PIR levels**			
<1.33	*Reference*		
1.33–3.5	0.989	(0.965–1.014)	0.383
≥3.5	0.985	(0.954–1.018)	0.362
Year cycle	0.996	(0.993–0.999)	0.038

*Weighted analysis was performed. OR, odds ratio; CI, confidence interval*.

### The Trends of Blood Lead Levels

**Figure 1:**
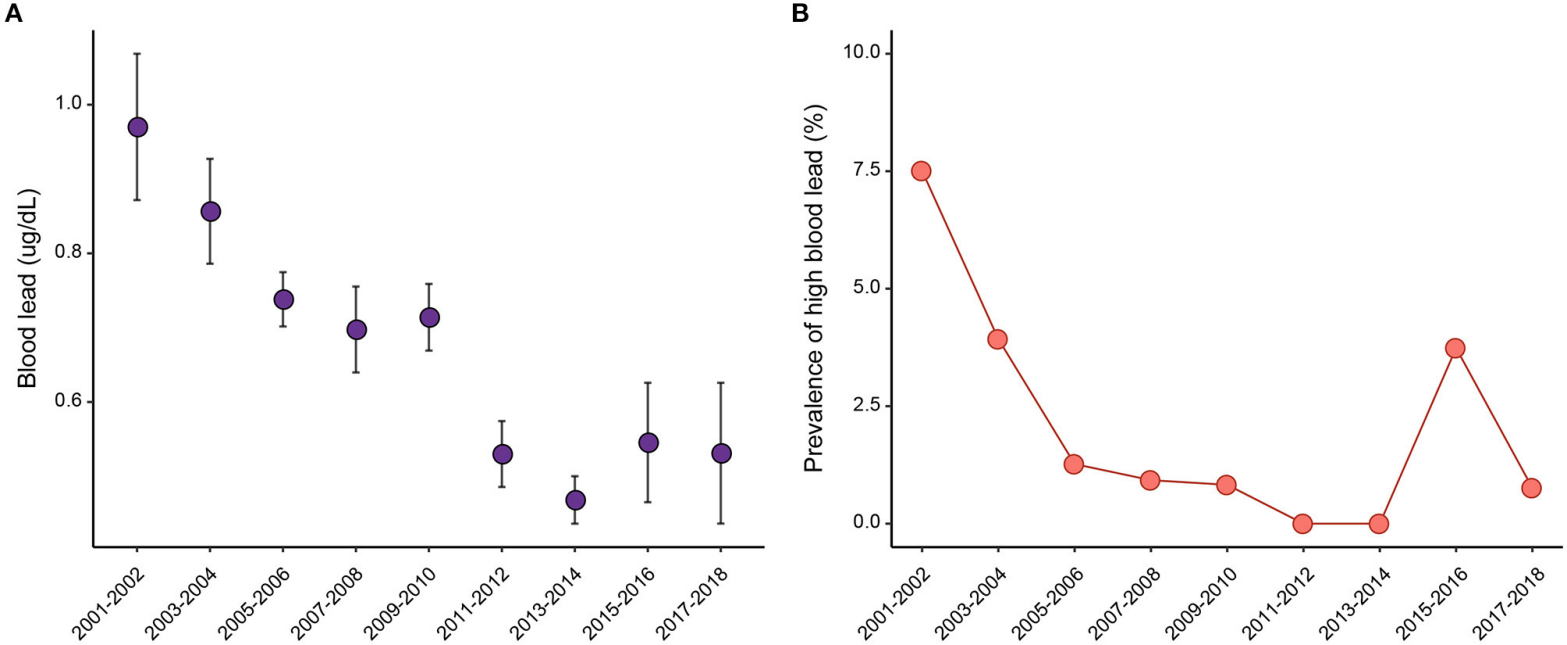
The **(A)** weighted blood lead levels and **(B)** prevalence of high blood lead for each survey period.

The mean blood lead level has decreased from 0.97 ug/dL in 2001–2002 to 0.46 ug/dL in 2013–2014 (Figure 1A; Supplementary Table 1). Afterward, a slight increase was observed with the mean blood lead of 0.55 ug/dL in 2015–2016 and 0.53 ug/dL in 2017–2018. Figure 1B shows similar trends in the prevalence of high blood lead. Before 2005, the prevalence rapidly dropped from 7.51% in 2001–2002 to 3.93% in 2003–2004. Then, the decreasing trend remained during 2005–2014 but with a slowed decline. However, the trend reversed after 2014, with a prevalence of 3.74% in 2015–2016 and 0.76% in 2017–2018.

**Figure 2:**
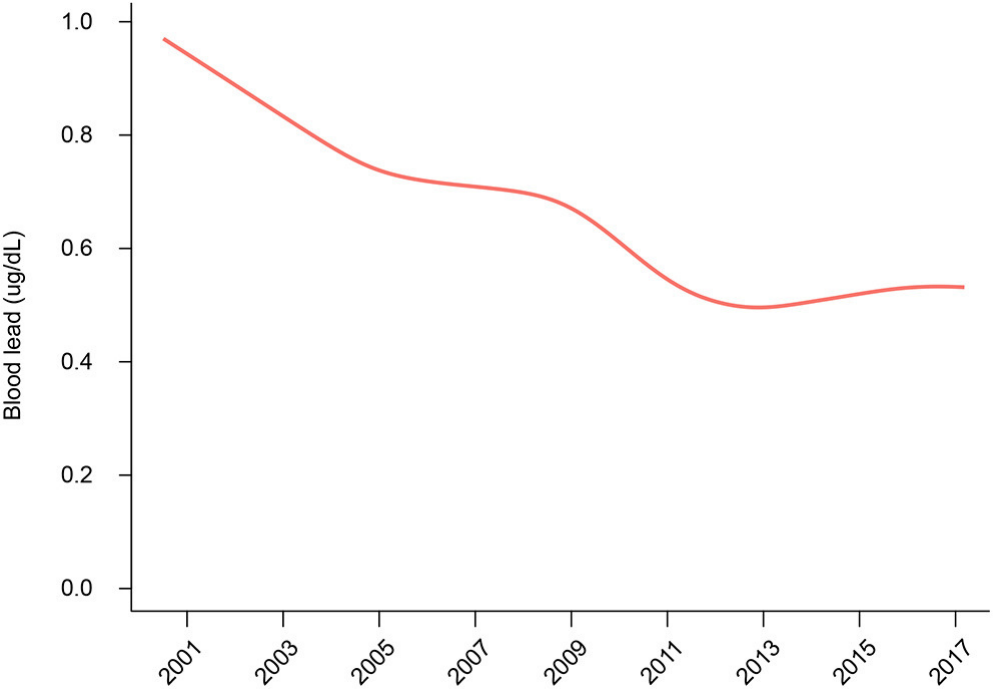
The smoothed curve on the association between year cycle and blood lead levels.

In Figure 2, we smoothed the association between year cycle and blood lead level, considering the 2-year cycles as a continuous variable. The smoothed curve showed that the blood lead declined with the year cycle in a stable trend during 2001–2012. However, when it comes to 2013, the curve became flat with a slightly increasing trend.

Table 3 summarizes the weighted linear regression for the time-related change in blood lead level in pregnant females. In the adjusted model (adjusted for age, race/ethnicity, education, and PIR levels), each year's increase was estimated to result in a 0.029 ug/dL decrease in blood lead. When analyzed as categorical variables, 2013–2014 showed an estimated 0.166 ug/dL decline compared with the 2009–2010 cycle (*P* = 0.005). In 2015–2016, the lead level showed a decreasing trend that nearly reached the statistical significance (*P* = 0.058). However, no significant change was observed in 2017–2018 cycle than 2009–2010 (β coefficient, −0.133, 95% CI, −0.346 ~ 0.080; *P* = 0.218). Additionally, when we set the lead level of 2013–2014 as a reference, the adjusted regression model showed no significant difference between 2015–2016 (*P* = 0.727) or 2017–2018 (*P* = 0.753) with 2013–2014.

**Table 3:** The weighted linear regression for the time-related change in blood lead level in pregnant females.

	**Crude model**			**Adjusted model**		
	**β coefficient**	**95% CI**	***P***	**β coefficient**	**95% CI**	***P***
Year	−0.029	(−0.041 ~−0.017)	<0.001	−0.029	(−0.041 ~−0.017)	<0.001
**Survey**						
2001–2002	0.256	(0.041 ~ 0.471)	0.02	0.294	(0.092 ~ 0.496)	0.005
2003–2004	0.143	(−0.023 ~ 0.309)	0.092	0.193	(0.019 ~ 0.367)	0.03
2005–2006	0.024	(−0.091 ~ 0.139)	0.677	0.088	(−0.026 ~ 0.201)	0.127
2007–2008	−0.016	(−0.162 ~ 0.129)	0.823	−0.03	(−0.165 ~ 0.104)	0.656
2009–2010	*Reference*			*Reference*		
2011–2012	−0.184	(−0.309 ~−0.059)	0.004	−0.114	(−0.241 ~ 0.014)	0.079
2013–2014	−0.246	(−0.355 ~−0.136)	<0.001	−0.166	(−0.280 ~−0.052)	0.005
2015–2016	−0.168	(−0.351 ~ 0.014)	0.07	−0.2	(−0.407 ~ 0.007)	0.058
2017–2018	−0.183	(−0.391 ~ 0.025)	0.084	−0.133	(−0.346 ~ 0.080)	0.218

*Adjusted for age, race/ethnicity, education, and PIR levels. Weighted analysis was performed. OR, odds ratio; CI, confidence interval*.

## Discussion

Lead is a neurotoxic metal naturally occurring in the earth's crust, which is soft, malleable, resistant to corrosion or fire, and able to absorb sounds or radiation. Owing to these characteristics, lead is widely used in many consumer products, and widespread lead exposure has become a global public health concern. For the general population, contaminated food, airborne lead, leaded plumbing pipes/fitting, and gasoline lead are common sources of lead exposure. Besides, some occupations are at a higher lead exposure level, such as lead miners, smelters, refiners, printers, battery manufacturers, and so forth (14, 15).

Lead exposure shows adverse effects on individuals of all ages, whereas pregnant women are particularly vulnerable with a higher risk of lead poisoning (16). The fetal bone formation elevates the demand for calcium and increases bone metabolic activity. The increased bone turnover would trigger lead release into the maternal blood, which makes an endogenous lead source during pregnancy (7–9). Epidemiologic and experimental data have revealed the potent reproductive and developmental toxicant of lead for both pregnant women and developing fetuses (17–19). Maternal lead exposure elevates the risk of multiple pregnancy complications, including anxiety (46), preeclampsia (20), gestational hypertension (21), spontaneous abortion, fetal growth restriction (22), and so forth (23–25). For the developing fetuses, lead can readily enter the developing brains and deleteriously affect the development of several organ systems (25, 26). Elevated lead levels could cause hearing problems, decrease intelligence quotient, and impair cognition/learning ability (27). Importantly, most adverse effects of lead poisoning (particularly adverse neurodevelopment) are irreversible and independent of postnatal lead exposure (26, 28).

Owning to the policies against environmental lead sources (i.e., gasoline, paint, plumbing pipes/fitting, and other consumer products), lead exposure has declined in the general US population (29–31). For the US women of childbearing age, the geometric mean blood lead has reduced from 10.37 to 0.61 ug/dL over the last four decades (1976–2016) (32). However, there lacks sufficient evidence for the blood lead levels in pregnant women. Considering the vulnerability of pregnant women and developing fetuses to the lead-related adverse effects, it requires particular attention to monitor the lead levels in this group. Based on a representative US population, our study showed that the weighted blood lead in pregnant women had declined about 45.36% (from 0.97 to 0.53 ug/dL) from 2001 to 2018. The decreasing trend in pregnant women is consistent with the general population. Based on a nationally representative study design, the estimated mean blood levels can be generalized to US pregnant women.

There is a widespread scientific consensus that the toxic effects of lead exposure affect maternal and fetus health across a wide range of exposures without a safe level limit (33, 34). It remains unclear at what exposure level the lead-related health risk begins to accumulate. No safe level of lead exposure has been identified in pregnant women, and the adverse effect was observed even at a lower exposure level previously considered harmless (35, 36). Previous studies have revealed lead toxicity at > 2 ug/dL (37–41). Still, using a dichotomous threshold is easy to interpret the trend of lead exposure over time compared with statistically derived cut points. Considering the low lead exposure in the US women of childbearing age, few participants showed a blood lead level of >5 ug/dL. Therefore, the currently recommended cut-off value might be insufficient to fully capture the picture of lead exposure in this population. This study defined blood lead > 2 ug/dL as high lead levels. Over 2001–2018, our study revealed a significant decrease in the prevalence of high blood lead from 7.51 to 3.74%.

Identifying high-risk pregnancy allows a better opportunity for the primary prevention of lead exposure, especially when there is a safe threshold for blood lead. A previous study analyzed determinants of blood lead in women of childbearing age based on the NHANES III (1988–1994) survey (35). Multiple adverse environmental factors associated with poverty and social injustice were revealed associated with lead exposure, including age, race/ethnicity, educational level, poverty, alcohol use, cigarette smoking, serum protoporphyrin level, and so forth (35). Our study also investigated the association between sociodemographic characteristics and blood lead level over 2001–2018. The Mexican American race was more likely to have higher lead levels, while no significant association was observed in age, education, and poverty. The difference in risk factors suggested that the role of sociodemographic disparities might have weakened. However, the results should be taken with care, and it does not mean that the regulation on lead content could be relaxed. Constant effort is still required to further control lead sources, especially for pregnant women who are of high gestational age, from high-risk communities, or low-income families (42–45).

The vulnerability of pregnant women to the lead-poisoning makes the surveillance of maternal lead exposure a serious public health priority. However, there is a lack of epidemiological data on the recent change in blood lead in pregnant women, and most primary prevention against lead exposure is currently focused on children. Also, recent evidence suggested that a high level of lead exposure still exists in women of childbearing age (32). Updated trends of blood lead would facilitate the future lead regulatory measures and increase health awareness for pregnant women. Over 2001–2018, we observed that the decreasing trend of blood lead in pregnant women seemed to slow down and even reversed since 2013. The weighted mean blood lead has increased from 0.46 ug/dL in 2013–2014 to 0.55 ug/dL in 2015–2016, and to 0.53 ug/dL in 2017–2018. The trends in pregnant women should be interpreted cautiously, and an overview of the current potential lead sources is required in the following research. Several reasons may explain the irregular trend. First, the mean age for first birth has grown from 24.9 years in 2000 to 26.3 years in 2014, and age is significantly associated with blood lead levels (46). The increased pregnancy age might offset the reduction in lead exposure. Second, pregnant individuals are younger and tend to have health-seeking behaviors compared with the general population. These characteristics make the subgroup individuals show a lower blood lead level (47). However, the slowing down trend suggested that the current health policies and guidelines against lead sources might be insufficient to reduce lead exposure further. Considering that very low lead exposure could impair cognition and mental development, continued monitoring is necessary for vulnerable individuals, such as children and pregnant women. Third, the slowing down trends indicated the remaining lead exposure in the environment and the potential unidentified lead sources. The following research is required to further investigate the potential lead exposure sources. Additionally, the NHANES survey was not designed for pregnant individuals, and the analysis focused on subgroups may potentially induce additional bias.

Lead exposure from industrial uses and environmental contamination has dramatically decreased over the past decades in developed countries. Still, diet remains one of the primary daily lead sources in developed countries, with leafy vegetables the most significant contributor (48). A low lead level was continuously detected in the food supply despite the substantial decrease in lead levels over decades. Lead in the environment could be settled on or absorbed by cereals, fruits, or vegetables, whereas the animals absorb lead via eating plants. It is impossible to completely remove lead from the environment or prevent lead from entering the food substances. Also, usual food processing steps can hardly remove lead content from plants or animals. However, due to the difference in food composition and processing conditions, it is difficult to quantify the diet-caused lead exposure in the population. Recently, Taylor and colleagues investigated the association between lead exposure and dietary patterns in pregnant women (48). The “all leafy green and green vegetables” pattern was more likely to have higher blood lead levels. Instead, a negative association was observed in the “confectionery” and “cakes and biscuits” dietary patterns (48). Interestingly, some nutrients could modify the absorption and excretion of lead. Vitamin D and phosphorus intake were positively associated with the bone lead in the middle- to elderly-aged men (48). In contrast, dietary calcium and iron intake were inversely associated with blood lead levels in pregnant individuals (49, 50).

Although intentional lead use has been banned in most developed countries, the lead content exists in many previously made products, which hardly biodegrade or disappear over time (51). The potential lead sources include lead-based paint and dust from houses built before 1978, and water provided by leaded plumbing pipes/fitting. Additionally, occupational and take-home exposures from the workplace are another preventable lead scouse (52). Since the revision of lead industry standards, blood lead levels in this population have significantly reduced (53).

Some limitations of this study should be noticed. First, NHANES collects a representative sample of the non-institutionalized US population. However, the number of pregnant people with valid blood lead levels was limited, which was insufficient to perform further subgroup analysis. Therefore, trends in blood level and their association with sociodemographic characteristics should be interpreted with caution. Still, this study provided population-based data on the trends of blood lead in US pregnant women. Second, the conclusion from this study might be unappropriated to generalize to other countries, especially developing ones. The blood lead level was higher in developing countries, and the lead scouse might be heterogeneous in different countries. Third, bone lead exposure was not analyzed in this study, suggesting the cumulative lead exposure for pregnant women. Further studies based on bone lead levels would be necessary. Forth, our results showed that the decrease in blood lead level seemed to slow down in US pregnant women. However, the underlying reasons remain unclear. Following studies should focus on the recent shifts in lead exposure scouse, improving regulatory measures for lead control.

## Conclusion

Based on the nationally representative data, this study summarized the trend of blood lead levels in US pregnant women from 2001 to 2018. Despite the substantial decline, the decreasing trend has slowed down since 2013. Continued effort is still required to control lead sources better and protect this population from lead exposure.

## Data Availability Statement

The original contributions presented in the study are included in the article/Supplementary Material, further inquiries can be directed to the corresponding authors.

## Ethics Statement

The studies involving human participants were reviewed and approved by National Center for Health Statistics Research Ethics Review Board. The patients/participants provided their written informed consent to participate in this study.

## Author Contributions

JW, YY, HL, HX, and NL designed the study and discussed the results and strategy. JW, YY, JZ, and HL performed analysis. HL, HX, and NL supervised, directed, and managed the study. All authors approved of the final version to be published.

## Conflict of Interest

The authors declare that the research was conducted in the absence of any commercial or financial relationships that could be construed as a potential conflict of interest.

## Publisher's Note

All claims expressed in this article are solely those of the authors and do not necessarily represent those of their affiliated organizations, or those of the publisher, the editors and the reviewers. Any product that may be evaluated in this article, or claim that may be made by its manufacturer, is not guaranteed or endorsed by the publisher.
