# Supplementary material for: Trends of Blood Lead Levels in US Pregnant Women: The National Health and Nutrition Examination Survey (2001–2018)
Source: Front Public Health. 2022 Jul 1;10:922563. doi: 10.3389/fpubh.2022.922563 (PMC9283785; doi:10.3389/fpubh.2022.922563)
Supplement: Supplementary file 1 [file Table_1.pdf]

Supplement Table 1. The blood lead from 2001-2002 to 2017-2018 cycle.

Cycle interval	Blood lead level (ug/dL)
2001-2002	0.97
2003-2004	0.86
2005-2006	0.74
2007-2008	0.70
2009-2010	0.71
2011-2012	0.53
2013-2014	0.47
2015-2016	0.55
2017-2018	0.53
